# Searching for a tactile target: the impact of set-size on the N140cc

**DOI:** 10.3389/fnhum.2023.1209555

**Published:** 2023-06-22

**Authors:** Elena Gherri, Fabiola Rosaria Fiorino, Cristina Iani, Sandro Rubichi

**Affiliations:** ^1^Dipartimento di Filosofia e Comunicazione, University of Bologna, Bologna, Italy; ^2^Department of Biomedical, Metabolic and Neural Science, University of Modena and Reggio Emilia, Modena, Italy; ^3^Department of Surgery, Medicine, Dentistry and Morphological Sciences With Interest in Transplant, Oncology and Regenerative Medicine, University of Modena and Reggio Emilia, Modena, Italy; ^4^Center of Neuroscience and Neurotechnology, University of Modena and Reggio Emilia, Modena, Italy

**Keywords:** touch, event-related potentials (ERP), selective attention, N140cc, tactile search, set-size

## Abstract

The time needed to find a visual target amongst distractors (search task) can increase as a function of the distractors’ number (set-size) in the search-array (inefficient search). While the allocation of attention in search tasks has been extensively investigated and debated in the visual domain, little is known about these mechanisms in touch. Initial behavioral evidence shows inefficient search behavior when participants have to distinguish between target and distractors defined by their vibro-tactile frequencies. In the present study, to investigate the allocation of attention to items of the search-array we measured the N140cc during a tactile task in which the set-size was manipulated. The N140cc is a lateralized component of event-related brain potentials recently described as a psychophysiological marker of attentional allocation in tactile search tasks. Participants localized the target, a singleton frequency, while ignoring one, three or five homogeneous distractors. Results showed that error rates linearly increased as a function of set-size, while response times were not affected. Reliable N140cc components were observed for all set-sizes. Crucially, the N140cc amplitude decreased as the number of distractors increased. We argue that the presence of additional distractors hindered the preattentive analysis of the search array resulting in increased uncertainty about the target location (inefficient preattentive stage). This, in turn, increased the variability of the deployment of attention to the target, resulting in reduced N140cc amplitudes. Consistent with existing behavioral evidence, these findings highlight systematic differences between the visual and the tactile attentional systems.

## 1. Introduction

The ability to select relevant information from a cluttered environment strongly depends on selective attention mechanisms. These mechanisms are typically investigated in search tasks in which participants have to identify the presence (or a feature) of a task relevant item (target) presented simultaneously with a number of irrelevant ones (distractors). Search efficiency is typically assessed by manipulating the number (set-size) and features of distractors presented with the target. Efficient search behavior is unaffected by set-size, as indicated by a flat slope function between performance indicators (response times and/or accuracy) and set-size and is usually observed when target and distractors can be distinguished on the basis of a distinctive feature (e.g., Egeth et al., 1972; Treisman and Gelade, 1980). The preattentive processing of all search-array items in parallel guides attention to the target (parallel search) (e.g., Wolfe, 1994, 2021). By contrast, inefficient search is characterized by increasingly worst performance as a function of set-size (e.g., Treisman and Gelade, 1980; Wolfe, 1994) and is often observed when target and distractors are highly similar (e.g., Duncan and Humphreys, 1989) or when they differ in terms of a combination of features (e.g., Treisman, 1991). Inefficient behavior, in terms of increase of RTs and errors as a function of set-size, has been suggested to reflect the serial allocation of attention to single items in the array (serial search) until the target is identified (e. g., Treisman and Gelade, 1980). However, this pattern of behavior can also be explained by a delayed shift of attention to the target due to longer preattentive processing of the array (*inefficient preattentive stage*, e.g., Folk and Remington, 1998) or by a longer attentional processing of the target (*inefficient attentional processing*, e.g., Christie et al., 2015).

While most studies on search behavior involved visual search tasks, researchers have started to address analogous questions in the tactile domain. When participants were allowed to haptically explore the tactile stimuli presented to their fingers, results revealed that search efficiency depended on the specific dimension defining target and distractors (c.f. Lederman and Klatzky, 1997; Overvliet et al., 2007). Efficient search was observed with abrupt surface discontinuity (e.g., deep vs. shallow hole) and material (e.g., roughness), while inefficient search was reported when participants searched for specific contours (e.g., slant vs. curve surface) and relative orientation (vertical vs. horizontal) (c.f. Lederman and Klatzky, 1997; Overvliet et al., 2007).

Other researchers have exploited the ability of the somatosensory system to discriminate the frequencies of different vibrotactile stimuli (e.g., Johansson and Vallbo, 1979; Bark et al., 2008). Groen et al. (2008) presented the array to the participants’ abdomen and manipulated both the set-size and the similarity between target and distractors. While search was inefficient on target present trials, no set-size effect was observed on target absent trials and no effect of target-distractor similarity was observed. Similar results were reported by Halfen et al. (2020) in a passive tactile search task in which vibrotactile stimuli were delivered to different parts of the body (hand, back, legs, etc.). Results showed set-size effects on RTs of both target-present and target-absent trials. Thus, in touch, search appears more inefficient on target present than on target absent trials, in contrast to evidence from the visual domain (e.g., Wolfe, 2021).

Event-related potential (ERP) studies have recently identified the electrophysiological correlate of target selection in tactile search tasks (e.g., Katus et al., 2015; Forster et al., 2016; Ambron et al., 2018; Katus and Eimer, 2019; Mena et al., 2020; Gherri et al., 2021, 2022). ERPs elicited by the search-array are typically more negative over the hemisphere contralateral to the target side compared to those observed over the ipsilateral hemisphere. This lateralized ERP component (labeled N140cc, N2cc, or CCN in different studies) is typically elicited over somatosensory areas from about 100–140 ms from the onset of the search-array (e.g., Forster et al., 2016). The presence of the N140cc in tactile search tasks was deemed particularly interesting due to the analogies with the N2pc component observed in visual search tasks (e.g., Luck and Hillyard, 1994; Eimer, 1996; Luck et al., 1997). The N2pc is considered an ERP marker of attentional allocation in the visual field and has been widely used to investigate the mechanisms underlying selective attention in visual search tasks over the last 30 years (for reviews see Luck, 2012; Eimer, 2014). Hence, recent studies have started to assess whether and to what extent the tactile N140cc can be considered the functional equivalent of the visual N2pc (Katus et al., 2015; Forster et al., 2016; Katus and Eimer, 2019; Mena et al., 2020; Gherri et al., 2021, 2022).

In a series of recent tactile search ERP studies, participants were asked to localize a target presented simultaneously with one salient but irrelevant singleton distractor and with several other homogeneous distractors (Mena et al., 2020; Gherri et al., 2021, 2022). On target-absent trials, an N140cc was elicited contralateral to the singleton distractor (Mena et al., 2020). On target-present trials, the amplitude of the target-elicited N140cc was reduced in the presence of a contralateral singleton distractor (Mena et al., 2020; Gherri et al., 2022). These observations suggest that the N140cc component reflects the allocation of tactile spatial attention to potentially task-relevant items in the array.

Notably, existing evidence has also shown potentially relevant differences between the mechanisms responsible for visual and tactile target selection. In the visual domain, the interference created by a singleton distractor increased as the distance between target and distractor decreases within the same hemifield, as revealed by worst performance and reduced N2pc components for contiguous singletons, due to the progressively overlapping neural resources assumed to represent these items (e.g., Hilimire et al., 2009, 2010; Gaspar and McDonald, 2014). In touch, however, performance improved (but the N140cc amplitude decreased) when the target and the singleton distractor were presented to contiguous fingers of the same hand (Gherri et al., 2021). This reveals systematic differences in the processes underlying target selection in the presence of a salient distractor between vision and touch.

**FIGURE 1:**
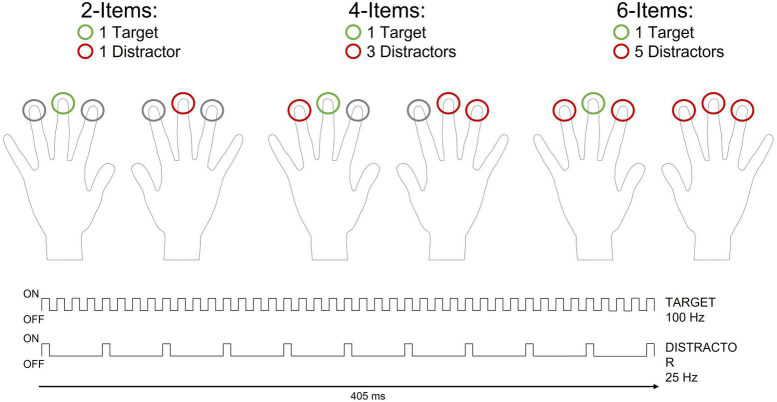
Schematic representation of the tactile search-array (405 ms duration) and the different types of set-sizes included in the present study. On each trial one target was presented to the index, middle or ring finger of the left or right hand (green circle). One, three, or five homogenous distractors were simultaneously presented with the target on different trials (red circles). The number of distractors varied randomly across trials. All search arrays included a symmetrical configuration of tactile stimuli over the left and right hand such that stimuli were always presented to mirror-symmetric (homologous) fingers of opposite hands. Target and distractors differed with respect to their vibrotactile frequencies. The target vibration (100 Hz) consisted of a sequence of pulses during which the rod was in contact with the skin for 5 ms (“ON”), followed by an inter-pulse interval of 5 ms (“OFF”) in which no contact was being made. For the distractor vibration vibrations (25 Hz), ON pulses of 5 ms were interleaved by OFF periods of 35 ms.

The studies reviewed above can be considered as a first attempt to characterize the functional meaning of the N140cc component and the mechanisms responsible for the selection of relevant information in touch. However, basic questions related to search efficiency and its impact on the N140cc component remain completely unexplored. In the visual modality, different search tasks have yielded different pattern of N2pc modulations by set-size, with some studies showing a decreasing N2pc with increasing distractors (e.g., Mazza et al., 2009; Tay et al., 2022), while others showing set-sizes effects on the N2pc duration (e.g., Christie et al., 2015). To investigate whether, similarly to the visual modality, set-size modulates the N140cc component elicited during a tactile search task, we asked participants to localize a target (on the left or right hand) while ignoring simultaneous distractors. In line with existing ERP studies, target and distractors differed with respect to their vibrotactile frequencies. On different trials, one, three or five homogeneous distractors were presented with the target (see Figure 1). In line with existing evidence, if performance in the search task is affected by set-size (c.f. Groen et al., 2008; Halfen et al., 2020), we expect to observe also a modulation of the N140cc amplitude.

## 2. Materials and methods

### 2.1. Participants

A total of thirty-seven volunteers were recruited via word-of-mouth at the University of Modena and Reggio Emilia. All of them had a normal or corrected-to-normal vision and no history of neurological disorders. Three participants were excluded from the analyses due to low accuracy levels in the behavioral task (overall accuracy levels across set-sizes below 60%). Following the ERP data processing procedure, six participants’ data were excluded due to a low number of trials (less than 60% of trials left after rejection of ERP artifacts for at least one of the set-size conditions). A total of 28 participants remained in the sample (23 females and 5 males, *M*_*age*_ = 21 years, *SD*_*age*_ = 3.6 years; 27 right handed and 1 ambidextrous, Oldfield, 1971).

This project was approved by the Area Vasta Emilia Nord (AVEN) Ethics Committee and followed the Helsinki Declaration principles. All participants signed an informed consent before starting the experiment.

### 2.2. Stimuli and apparatus

Participants placed their hands on a table with their palms down. Tactile stimuli were presented using 12 V solenoids (Heijo Research Electronics, London, UK) driving a metal rod with a blunt conical tip. The tip of the tactile stimulators touched the skin whenever a current passed through the solenoid. Six tactile stimulators were used in total, each one was attached with adhesive medical tape to the inner side of the top phalanx of the left and right index, middle and ring fingers. The distance between the index fingers of the two hands was 10 cm. Once in the correct position, the hands were covered with a black cardboard, on top of which there was a white pin aligned with the body midline which served as fixation-point. To mask the sounds made by tactile stimulators, one speaker was positioned on the table close to the hands and presented white noise (65 dB SPL) throughout the experimental blocks. Two vertically arranged foot-pedals (top and bottom pedals) were positioned under the toes and heel of one of the participants’ feet. Participants were asked to keep one foot on these response pedals during the task.

On each trial, a search-array was presented to mirror-symmetric (homologous) fingers of the left and right hand. There were three different types of search-arrays in which two, four or six vibro-tactile stimuli were presented simultaneously (2-Items, 4-Items and 6-Items arrays, respectively, see Figure 1). One target stimulus was presented on each trial, while the number of distractor(s) varied on different trials (1, 3 or 5 distractors). Vibro-tactile stimuli differed with respect to their vibration frequencies (25 Hz or 100 Hz). The target was the fastest vibration (100 Hz), while the distractors were the slowest vibrations (25 Hz). These frequencies consisted of a rapid sequence of pulses during which the rod was in contact with the skin for 5 ms, followed by a variable inter-pulse interval set at 35 ms and 5 ms for the slow and fast vibrations, respectively (Figure 1). All stimuli had a total duration of 405 ms. The tactile search-array started and ended with all the stimulators touching the skin simultaneously to prevent participants from using the offset of the stimuli to complete the task.

### 2.3. Procedure

Each trial started with a 300 ms empty interval which was followed by the simultaneous presentation of the tactile target and of the distractor(s) (405 ms duration). The search-array presentation was followed by a 1,800 ms interval which was used to collect foot responses. Thus, the interstimulus interval between search-arrays was set at 2.505 ms.

Ten blocks of 72 trials each were completed by participants. The tactile target was presented randomly and with equal probability to the index, middle or ring finger of the left or right hand (12 trials for each target location in each block). Within each block of trials, 2-, 4-, and 6-items search-arrays were equally likely (each presented on 24 trials per block).

Participants’ task was to identify the location of the target (left or right hand) by pressing the top or bottom pedal with their toes or heels. They were instructed to keep their eyes on the central fixation at all times and to press the pedals as fast and as accurately as possible. To eliminate lateralized motor activity in the grand averaged ERP waveforms, participants completed five successive blocks responding with their right foot, and the remaining five blocks with their left foot. The order of the responding foot was counterbalanced across participants.

Prior to the beginning of the experiment participants completed two 36-trials practice blocks (which was repeated whenever average accuracy fell below 60% during this training), following a familiarization procedure with the stimuli frequencies. At the end of each block participants received verbal feedback about their performance (average response time and accuracy).

### 2.4. Electroencephalography recording and data analysis

Electroencephalography (EEG) was recorded with a BrainAmp amplifier system (500 Hz sampling rate) from 64 active electrodes positioned according to 10–20 system. EEG data was analyzed using Brain Vision Analyser (version 2.0.4.368). EEG was digitally re-referenced to the average of the left and right earlobe and was digitally filtered offline (high-pass filter 0.53 Hz, low-pass filter 40 Hz and notch filter 50 Hz). The EEG was epoched into 450 ms intervals starting 100 ms before and ending 350 ms after the search-array onset. Trials with eye blinks, horizontal eye movements and other artifacts (voltage exceeding ± 80 μV at any electrode sites) were excluded from further analysis, as were trials with response errors. Participants with less than 60% of the trials in at least one of the set-size conditions were excluded from the analyses. This led to the exclusion of 6 participants. The average number of trials included in each condition for the remaining 28 participants was 185 for the 2-items array (77% of these trials), 167 for the 4-Items (70%) and 150 for the 6-Items (63%).

Event-related potentials elicited by the presentation of the search array on correct trials were averaged relative to a 100 ms pre-stimulus baseline separately for all combinations of set-sizes (2-Items vs. 4-Items vs. 6-Items search-array) and target side (left vs. right hand). The N140cc was quantified for each participant and for each set-size on the basis of ERP mean amplitudes obtained at lateral central electrodes C3/4 and C5/6 (where this component was maximal in the present study, in line with previous studies from our lab, Gherri et al., 2021) over the hemisphere contralateral and ipsilateral to the target side in the 110–250 ms post-array onset measurement window (see Gherri et al., 2021, 2022). To investigate whether set-size modulated the amplitude of the N140cc component, a repeated-measures analysis of variance (ANOVA) was conducted on the pooled mean amplitude values measured at electrodes pairs C3/4 and C5/6 for the factors set-sizes (2-Items vs. 4-Items vs. 6-Items search-array) and laterality (hemisphere contralateral vs. ipsilateral to the target side). In these analyses, the presence of reliable lateralized components is reflected by the main effect of the factor laterality, indicating significant differences between the contralateral and ipsilateral hemispheres to the target side. Following significant laterality x set-sizes interactions, separate analyses were carried out for each set-size, to determine the presence of reliable N140cc lateralized components. Then, the difference waveforms between contralateral and ipsilateral ERPs were calculated, separately for the different set-sizes. To determine whether the N140cc amplitude was modulated by set-size, we run contrasts (by means of *t*-tests) between these difference waveforms observed for the different set-sizes (Bonferroni corrections were applied to these multiple comparisons). Finally, to test whether the N140cc amplitude was linearly related to the set-size of the search-array, polynomial orthogonal contrasts were carried out.

While the method of choice to measure the onset and offset of a lateralized component is the fractional peak latency obtained from jackknife-averaged ERPs (e.g., Miller et al., 1998; Ulrich and Miller, 2001; Kiesel et al., 2008) within the time window of interest (110–250 ms), the lack of clear peaks in the N140cc components makes this approach prone to distortions. Thus, to examine the time course of the target-elicited N140cc in the different search arrays, the mean N140cc amplitudes were also analyzed in 20 ms consecutive time windows starting 90 ms and terminating 310 ms post-array onset, separately for each set-size (e.g., McDonald et al., 2013; Christie et al., 2015). Results are shown in Table 1. In these analyses, for any three consecutive 20 ms time windows at least two had to reveal significant effects to indicate the reliable presence of the N140cc components.

**TABLE 1:** Comparisons between ipsilateral and contralateral waveforms computed separately for the three set-sizes for consecutive 20 ms time-windows, from 90 to 310 ms post-array onset.

20 ms time-windows	2-Items t (*p*-value)	4-Items t (*p*-value)	6-Items t (*p*-value)
90–110	−2.402 (0.023)*	−0.620 (0.540)	−0.833 (0.412)
110–130	−4.894 (0.001)*	−1.979 (0.058)	−0.958 (0.347)
130–150	−6.714 (0.000)*	−2.943 (0.007)*	−0.124 (0.019)*
150–170	−8.380 (0.000)*	−3.788 (0.001)*	−1.905 (0.067)
170–190	−5.820 (0.000)*	−3.856 (0.001)*	−3.333 (0.003)*
190–210	−5.982 (0.000)*	−3.507 (0.002)*	−2.975 (0.006)*
210–230	−5.707 (0.000)*	−4.374 (0.000)*	−3.197 (0.004)*
230–250	−5.653 (0.000)*	−2.926 (0.007)*	−2.079 (0.047)*
250–270	−2.379 (0.025)*	−0.791 (0.436)	−1.541 (0.135)
270–290	−1.262 (0.218)	−0.770 (0.448)	−2.002 (0.055)
290–310	0.336 (0.740)	−0.998 (0.327)	−1.990 (0.057)

Each cell shows the values of this *t*-test and the corresponding *p*-value in brackets. Asterisks denote the presence of a significant lateralization (*p* < 0.05). In these analyses, for any three consecutive 20 ms time windows at least two had to show significant effects to indicate the reliable presence of the N140cc components (highlighted in gray).

In addition to the planned analyses of the N140cc, an exploratory analysis was also performed on an earlier lateralized component emerged between 70 and 100 ms post-array onset. Figures 3, 4 clearly show this early lateralization overlapping with the N80 component, characterized by an amplitude modulation by set-size analogous to the one observed for the N140cc. To further quantify the statistical reliability of this early lateralization and its amplitude modulation by set-size, mean amplitude values obtained between 70 and 100 ms post-array onset were analyzed following the same analysis procedure adopted for the N140cc.

Mean RTs were calculated on correct response times trimmed to 2.5 standard deviations from the mean (calculated separately for each participant and each set-size). Mean reaction times (RTs) as well as accuracy rates were submitted to separate repeated measure ANOVAs with set-size (2 Items vs. 4 Items vs. 6 Items search-array) as within-subjects factor. Following set-sizes main effects, we run contrasts between the means observed for the different set-sizes (Bonferroni corrections were applied to these multiple comparisons). Similarly to the ERP analysis, polynomial orthogonal contrasts were also carried out to investigate whether mean RTs and accuracy rates were linearly dependent on set-size.

For all analyses, Greenhouse-Geisser adjustments to the degrees of freedom were applied where appropriate, and unadjusted *p*-values were reported.

## 3. Results

### 3.1. Behavioral results

**FIGURE 2:**
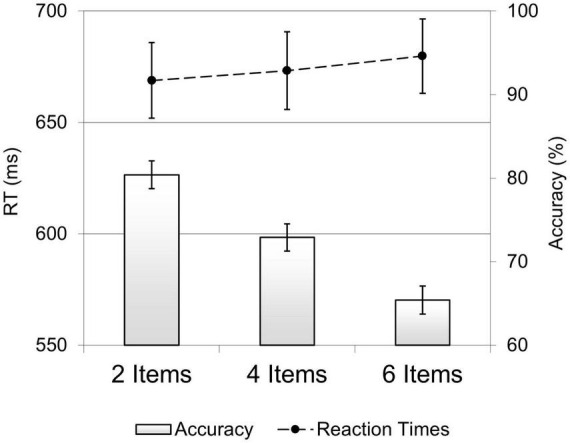
The effect of set-size on behavioral performance. Accuracy rates (bar graph) and mean RTs (line graph) in the 2, 4, and 6 items set-size trials. Error bars represent the standard errors of the means.

For the accuracy data (Figure 2, bar graph), the ANOVA showed a main effect of set-size, *F*(1.74, 46.9) = 95.9, *p* < 0.001, η_*p*_^2^ = 0.78, with participants’ performance being most accurate in the 2 Items array (*M* = 80.4%, SE = 1.7), intermediate in the 4 Items array (*M* = 73%, SE = 1.6) and worst in the 6 Item arrays (*M* = 65%, SE = 1.7) (all Bonferroni adjusted contrasts, *p* < 0.001). Accuracy rates linearly decreased with increasing set-sizes, *F*(1,27) = 144, *p* < 0.001, η_*p*_^2^ = 0.84.

Results of the ANOVA carried out on RTs revealed no significant effect of set-size, *F*(1.38,37.2) = 1.7, *p* = 0.189, η_*p*_^2^ = 0.06, see Figure 2 (line graph).

### 3.2. ERP results

**FIGURE 3:**
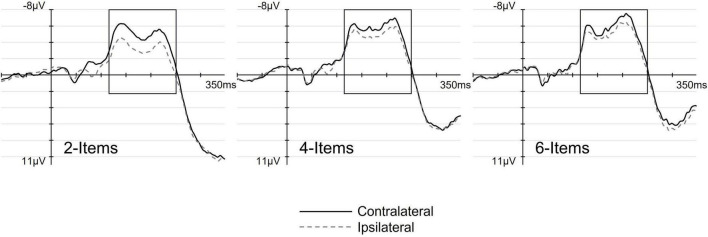
The effect of set-size on the N140cc lateralized component. Panels show ERPs elicited over pooled electrodes C3/4 and C5/6, contralateral (solid line) and ipsilateral (dashed line) to the target side, separately for the 2-, 4-, and 6-Items search-arrays.

**FIGURE 4:**
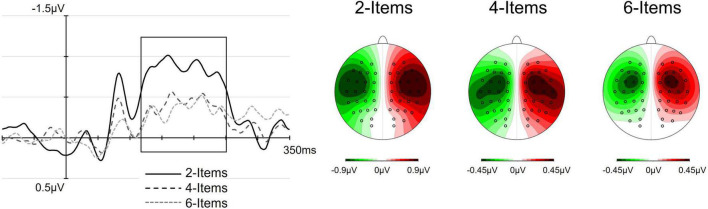
The effect of set-size on the N140cc lateralized component. **(Left)** panels show the difference waveforms (contralateral–ipsilateral waveforms) for pooled electrodes sites C3/4 and C5/6. In these figures, the black lines represent the N140cc amplitude in the 2-items array, the dark gray lines (long dash) represent the 4-items array and the light gray line (short dash) represent the 6-item array. The box shows the time window considered for statistical analyses (110–250 ms). **(Right)** panels show the scalp distribution of the lateralized N140cc component measured in the 110–250 ms interval, separately for each set-size.

Figure 3 shows ERP waveforms elicited at pooled electrodes C3/4 and C5/6 contralateral (solid lines) and ipsilateral (dashed lines) to the location of the tactile target, separately for each set-size. The corresponding difference waveforms (Figure 4) were obtained by subtracting ERPs elicited at electrodes ipsilateral to the target from contralateral ERPs. As can be seen from both Figures 3, 4, the lateralized N140cc component was present approximately between 110 and 250 ms post array onset and appeared to be modulated by set-size. Interestingly, the N140cc was preceded by an earlier lateralization between 70 and 100 ms post array, similarly modulated by set-size. The scalp distribution of the lateralized components observed in the 110–250 ms post-array interval is shown in Figure 4.

#### 3.2.1. Early lateralization (70–100 ms)

An exploratory analysis of the mean amplitude values measured between 70 and 100 ms post array onset showed that the main effect of set-size was not statistically significant, *F*(1.9, 50.9) = 1.06, *p* = 0.35, η_*p*_^2^ = 0.038, suggesting no differences between ERPs elicited by the different set-sizes. The main effect of the factor laterality, *F(1,27)* = 18.2, *p* < 0.001, η_*p*_^2^ = 0.43, revealed the presence of a reliable lateralized component between 70 and 100 ms regardless of set-size. This was further modulated by set-size (laterality × set-size interaction), *F(1.7,45)* = 5.19, *p* < 0.013, η_*p*_^2^ = 0.16. Pair-wise comparisons between ipsilateral and contralateral ERP amplitudes carried out separately for each set-size revealed the presence of reliable lateralizations for the 2-items array [*t*(27) = 4.97, *p* < 0.001, *d* = 0.9] and 4-items array [*t*(27) = 2.95, *p* < 0.006, *d* = 0.6] but not for the 6-items array [*t*(27) = 1.47, *p* = 0.15, *d* = 0.3]. The amplitude of this early lateralized component linearly decreased as a function of set-size [*F*(1,27) = 8.1, *p* < 0.008, η_*p*_^2^ = 0.23]. Bonferroni-adjusted contrasts carried out between the amplitudes observed for the different set-sizes showed a larger lateralized component for 2- Items arrays compared to 4- and 6-Items arrays (both *p* < 0.033). However, no statistical difference emerged between 4- and 6-Items arrays (*p* = 0.9).

#### 3.2.2. N140cc (110–250 ms)

The planned analysis of mean amplitude values (measured in the 110–250 ms interval) revealed a significant main effect of set-size, *F(1.7,47.2)* = 5.6, *p* < 0.006, η_*p*_^2^ = 0.17, that was driven by the fact that ERP amplitudes increased with set-size regardless of laterality. The N140cc component was reliably present between 110 and 250 ms (main effect of the factor laterality, *F(1,27)* = 40.02, *p* < 0.001, η_*p*_^2^ = 0.6). Crucially, the interaction of interest between laterality and set-size was significant, *F(1.59,42.8)* = 13.1, *p* < 0.001, η_*p*_^2^ = 0.33. Pairwise comparisons between ipsilateral and contralateral ERP amplitudes carried out separately for each set-size revealed the presence of reliable lateralized N140cc components for each set-size (all *t*(27) > 2.9, *p* < 0.007, *d* > 0.055). The amplitude of the N140cc component linearly decreased as a function of set-size, *F*(1,27) = 17.6, *p* < 0.001, η_*p*_^2^ = 0.39. Bonferroni-adjusted contrasts carried out between the N140cc amplitude observed for the different set-sizes showed a larger N140cc for 2- Items arrays compared to 4 and 6 Items arrays (both *p* < 0.001). However, no statistical difference emerged between 4 and 6 Items arrays (*p* = 0.6).

To evaluate the time course of the N140cc we computed the mean amplitude values of the waveforms ipsilateral and contralateral to the target separately for each set-size in consecutive 20 ms intervals from 90 to 310 ms (see Table 1). Reliable N140cc emerged in the 2-Items array from 90 to 270 ms post-array. The N140cc was observed in the 4-Items array between 130 and 250 ms, while it was present between 170 and 250 ms in the 6-Items array.

## 4. Discussion

Within the more general purpose of understanding similarities and differences between the neural mechanisms responsible for the allocation of attention in the visual and tactile sensory domains, the aim of this study was to investigate whether the amplitude of the N140cc component elicited during a tactile search task is modulated by set-size. Participants showed inefficient search behavior, as indicated by the fact that accuracy rates linearly decreased as a function of distractors’ number, although RTs were not affected by set-size. The N140cc component was reliably present contralateral to the target side regardless of the number of distractors. Crucially, the amplitude and time course of the N140cc component was modulated by set-size. The N140cc amplitude was maximal and emerged in earlier time-windows in the 2-items array and decreased linearly as the number of distractors increased.

According to Wolfe’s (1994) Guided Search model, an activation map is created upon the presentation of the search-array. The location of the target is characterized by the largest activation in the map and attention is subsequently shifted toward this location. When the target pops out from the array, its identification is not hindered by increasing distractor numbers (efficient search) and does not require further attentional processing. By contrast, when the target-defining feature is relatively subtle, the quality of this pre-attentive processing can be affected by the distractor number and an in-depth attentional processing may be necessary for the target identification.

In the present study, the number of distractors reduced the accuracy of target localization showing that the target did not pop out from the array despite being more salient than the homogenous distractors (c.f. Groen et al., 2008; Halfen et al., 2020). This is further supported by the presence of reliable N140cc for each set-size which confirms that attention was shifted to the target side for in-depth attentional processing. Because the N140cc is assumed to be time-locked to the shift of attention toward the target location (Mena et al., 2020), ERP differences between the different set-sizes can help to interpret the behavioral inefficiency observed in the present study. In search accuracy experiments, set-size effects are assumed to be directly related to capacity limits on the quality of processing (c.f. Shaw, 1984; Palmer et al., 1993). We argue that the manipulation of set-size already affected the preattentive analysis of the search array, leading to weaker activations of the target location on the map when additional distractors flanked the target. In turns, this reduced attentional guidance for larger set-sizes affected the attentional processing of the target as shown by reduced and delayed N140cc components. This increased uncertainty about the target location resulted in delayed or more variable times when attention was shifted and in an increased number of attention shifts to either side of the array. Due to the averaging process across trials, this resulted in reduced N140cc amplitudes. In other words, weaker target location activations in the preattentive map for larger set-sizes gave rise to increasingly unguided search patterns, in line with previous N2pc studies that failed to observe a reliable component when the target did not pop out from the array (e.g., Dowdall et al., 2012)^1^. Thus, the present ERP findings show inefficiencies at both the preattentive and attentive stages of processing^2^.

Intriguingly, in addition to the N140cc components, an early lateralization was elicited between 70 and 100 ms over central electrodes and modulated by set-size, with decreasing amplitudes for larger set-sizes. We speculate that this early component may reflect the efficiency of the preattentive analysis, similarly to the PPC component observed in visual search tasks when a singleton item is presented amongst homogeneous distractors (e.g., Jannati et al., 2013). If this is the case, this would offer further evidence for systematic differences between set-sizes during the preattentive stage of processing, before the onset of the N140ccc. While this is an interesting possibility, it is crucial that future studies further investigate the presence and the functional meaning of this lateralized component which was observed here for the first time to our knowledge.

It is worth noting that the increased perceptual noise experienced by participants with larger set-sizes may be due - at least in part - to effects of tactile masking which are known to cause distortions in the sensory representation of the tactile stimulus (e.g., Gilson, 1969). In the present study the vibrotactile frequencies chosen for target and distractor (100 Hz and 25 Hz, respectively) were specifically aimed at activating different somatosensory channels (Pacini corpuscules, for high frequency perception, >40 Hz; Rapidly adapting fibers, Meissner corpuscules for lower frequency perception, <40 Hz, respectively; e.g., Gescheider et al., 2010) with the aim of reducing fusion effects driven by shared encoding mechanoreceptors. However, recent evidence has shown that these masking effects can also be observed across different somatosensory channels and different hands (Kuroki et al., 2017). It is therefore possible that participants experienced some masking of the vibrotactile frequencies and that this masking increased with larger distractor numbers. While this study represents a first attempt to track the physiological correlates of target selection in a tactile search task as a function of set-size, future studies should systematically aim at assessing the impact of masking on the attentional strategies adopted to select the target.

The pattern of decreasing N140cc amplitude observed in the present study differs substantially from those reported in visual search studies investigating the N2pc modulation by set-size. Mazza et al. (2009) observed that the N2pc amplitudes increased as a function of set-size regardless of whether behavioral results showed efficient or inefficient searches. It was argued that the larger N2pc amplitudes reflected the greater need for target enhancement in the presence of multiple distractors. Other studies have also reported an increase in the amplitude of the N2pc when additional distractors were present in the search-array (Luck and Hillyard, 1994; Eimer, 1996; Luck et al., 1997; Salahub and Emrich, 2018; Tay et al., 2022). However, there is also evidence that set-size does not necessarily modulate the N2pc amplitude. In an inefficient visual search task in which all items in the array were color singletons, set-size was found to modulate the *duration* of the N2pc (Christie et al., 2015). The time needed to process a subtle target feature increased when the display contained additional distractors (inefficient attentional processing). The absence of N2pc onset time modulations suggested that attention was directed to the target at the same time regardless of set-size (Christie et al., 2015). By contrast, in a different inefficient task (find an O amongst Cs) no effect of set-size was observed on the N2pc and no N2pc was elicited when all trials were averaged together (Dowdall et al., 2012). However, when these were sorted according to the N2pc latency a small N2pc emerged on faster trials (Dowdall et al., 2012). This N2pc latency variability was interpreted as evidence for a serial allocation of attention to different items of the array on different trials. Together, the studies discussed above clearly show how the attentional selection of the target is strongly dependent on the specific search task used and how similar (efficient or inefficient) patterns of behavioral results can be associated with different patterns of electrophysiological correlates of target selection.

One important difference between the N140cc and the N2pc is related to the time course of these components. While the N2pc is characterized by a sharp onset, an equally abrupt offset and a short duration (which can be measured through the jackknife procedure, Ulrich and Miller, 2001), the N140cc has a very shallow onset and offset and typically lasts for several tens of millisecond. Hence, it is particularly difficult to determine the exact time course of this component. It is possible that this smeared appearance of the N140cc is due to the specific stimuli used so far in ERP studies on tactile search given that the perception of vibrotactile frequencies unfolds over time, with stimulation periods alternated by non-stimulation ones. This is likely to increase the time jitter of the attentional deployment. Given the limitations related to the time course measurement of the N140cc, it is perhaps not surprising that existing studies have often reported a link between the amplitude of the N140cc and the accuracy observed in the tactile search task (Ambron et al., 2018; Gherri et al., 2021, 2022). These findings together with the results of the present study suggest that the amplitude of the N140cc may reflect the certainty (or systematicity) with which participants deploy attention to the target location within the search-array. One question that remains unexplored is whether similar properties of the N140cc can also be observed in tactile search tasks in which the target-defining feature is temporally discrete (i.e., does not change over time, line orientation, shape, etc., instead of a vibrotactile frequency). Future studies should investigate whether target selection in touch is a process intrinsically more variable across trials or whether this depends on the specific search tasks used so far.

To conclude, the present study investigated for the first time whether the N140cc component elicited in a tactile search task is modulated by set-size. The target and the homogeneous distractors differed with respect to their vibro-tactile frequencies. Results showed that both accuracy and the N140cc amplitude decreased linearly as the items in the search-array increased. We suggest that this pattern of results reflects inefficiencies already during the preattentive analysis, caused by the increasing noise that additional distractors create in the perceptual landscape. While attention is on average directed to the target side regardless of set-size, the increased number of distractors decreases attentional guidance and the certainty with which the target can be identified, resulting in reduced N140cc components.

## Data availability statement

The raw data supporting the conclusions of this article will be made available by the authors, without undue reservation.

## Ethics statement

The studies involving human participants were reviewed and approved by the Area Vasta Emilia Nord (AVEN) Ethics Committee. The patients/participants provided their written informed consent to participate in this study.

## Author contributions

EG ideated the study. SR, CI, and FF contributed to the design and implementation of the research. FF programmed the experiment and collected the data. EG and FF analyzed the data and wrote a first draft of the study. CI and SR reviewed and edited the manuscript. All authors provided critical feedback, helped shape the research and the manuscript, and read and agreed to the published version of the manuscript.

## Footnotes

1 An inefficient attentive process should lead to inefficient behavior. In the present study, we observed increasing errors but no RT modulations with increasing set-size. We believe that the lack of an effect on RTs was caused by the brief duration of the search array (400 ms) which was necessary to limit artifacts and eye movements in the present ERP study. However, previous behavioral studies using similar tactile search tasks (based on vibrotactile frequencies) found strong RT modulations as a function of set-size with longer search array durations (2,000 ms) (c.f. Groen et al., 2008; Halfen et al., 2020). The average RT in the present study was approximately 680 ms, thus participants selected and executed the response soon after the search array offset. Because frequency discrimination is complex and unfolds over time, it is possible that they were unable to maintain a mnemonic representation of the array for long after the array offset and terminated the search soon after stimuli disappeared even when they were still uncertain about the target location.

2 Alternatively, the reduced N140cc amplitudes observed with larger set-sizes could reflect a stronger attenuation of neural activity driven by the distractors (e.g., Luck et al., 1997 for the *filtering hypothesis* of the N2pc), rather than the selection/enhancement of the target (*target processing hypothesis*, e.g., Mazza et al., 2009). However, while the filtering hypothesis can account for the results of the present study, it is not consistent with studies showing that the N140cc is not affected by increased filtering demands when a salient singleton distractor is presented next to the target (e.g., Mena et al., 2020; Gherri et al., 2021).
